# The Role of Mast Cells in Autoimmune Bullous Dermatoses

**DOI:** 10.3389/fimmu.2018.00386

**Published:** 2018-02-28

**Authors:** Xinhua Yu, Anika Kasprick, Karin Hartmann, Frank Petersen

**Affiliations:** ^1^Priority Area Asthma and Allergy, Research Center Borstel, Borstel, Germany; ^2^Airway Research Center North (ARCN), German Center for Lung Research (DZL), Borstel, Germany; ^3^Lübeck Institute of Experimental Dermatology, University of Lübeck, Lübeck, Germany; ^4^Department of Dermatology, University of Lübeck, Lübeck, Germany

**Keywords:** mast cells, autoimmune bullous dermatoses, mouse models, autoantibodies, pathogenesis

## Abstract

Skin mast cells (MCs), a resident immune cell type with broad regulatory capacity, play an important role in sensing danger signals as well as in the control of the local immune response. It is conceivable to expect that skin MCs regulate autoimmune response and are thus involved in autoimmune diseases in the skin, e.g., autoimmune bullous dermatoses (AIBD). Therefore, exploring the role of MCs in AIBD will improve our understanding of the disease pathogenesis and the search for novel therapeutic targets. Previously, in clinical studies with AIBD, particularly bullous pemphigoid, patients’ samples have demonstrated that MCs are likely involved in the development of the diseases. However, using MC-deficient mice, studies with mouse models of AIBD have obtained inconclusive or even discrepant results. Therefore, it is necessary to clarify the observed discrepancies and to elucidate the role of MCs in AIBD. Here, in this review, we aim to clarify discrepant findings and finally elucidate the role of MCs in AIBD by summarizing and discussing the findings in both clinical and experimental studies.

## Introduction

Autoimmune bullous dermatoses (AIBD) are a group of autoimmune disorder affecting structure proteins in the skin which either mediate cell–cell or cell–matrix adhesion (1). Autoantibodies directed against these proteins impair the adhesion resulting in skin split formation and thus in a loss of the barrier integrity (1). Based on the location of split formation, AIBD can be categorized into two subgroups, pemphigus and pemphigoid disorders. Pemphigus diseases are intraepidermal blistering diseases caused by autoantibodies against adhesion molecules of the epidermis, such as desmoglein 1, desmoglein 3, envoplakin, and periplakin (1). In contrast to pemphigus, pemphigoid diseases are featured by subepidermal split formation (2).

Mast cells (MCs) originate from precursors of the hematopoietic lineage in the bone marrow (3). After homing to their target tissues, the precursors develop into mature MCs, a process regulated by various factors, including adhesion molecules and several cytokines. Among these, stem cell factor (SCF), a ligand of KIT, represents the most important one (4). MCs are distributed throughout almost all tissues and reside in proximity to nerves, blood, and lymphatic vessels (5). Besides the most prominent high-affinity receptor for IgE (FcεRI), MCs also express a plethora of other receptors, e.g., complement receptors, FcγR, and toll-like receptors enabling a response toward diverse stimuli (5, 6). Upon activation, MCs release a wide spectrum of products, including immediately secreted mediators like histamine and proteases, rapidly synthesized bioactive metabolites derived from arachidonic acid, such as prostaglandins and leukotrienes, and newly synthesized molecules *via* upregulated gene expression in response to stimulation, including most cytokines and chemokines (5). Their wide tissue distribution and broad activating capacity designates MCs as major immune cells at the body interface regulating both innate and adaptive immunity (7, 8). These properties give rise to the question whether MCs have the ability to regulate immune responses against autoantigens and in skin autoimmune diseases like AIBD. This idea has been substantiated by clinical observations in which MC activation has been shown in some AIBD, and experimentally by employing MC-deficient mice in animal models of AIBD. In this mini-review article, we summarize and discuss clinical and experimental findings to clarify the role of MCs in these diseases.

## MCs in Human AIBD

The first evidence indicating a role of MCs in AIBD can be traced back to 1978, when Wintroub et al. investigated bullous pemphigoid (BP) (9), the most common autoimmune blistering disease characterized by autoantibodies against the autoantigens BP180 (also named type XVII collagen, COL17) and BP230 (10). Besides autoantibodies and complement deposition at the basement-membrane zone, they observed a progressive MC degranulation and subsequent eosinophil infiltration in affected skin of BP patients (9). This MC degranulation was associated with elevated levels of MC-derived mediators and proteases, including eosinophil chemotactic factor, in bullous fluid suggesting for the first time a role of MCs in the pathogenesis of BP (9). Later, Dvorak et al. confirmed this hypothesis by investigating histological changes of clinical lesions in a BP patient during pathogenesis (11). They found that the clinical lesions were featured by a sequence of histopathologic events starting with MC degranulation and proceeding to infiltration of lymphocytes and later on eosinophils and basophils. The notion of a role of MC in BP was further strengthened when Delaporte et al. discovered that most patients express IgE autoantibodies which specifically activate MCs and eosinophils (12). In 2007, Fairley transferred total IgE isolated from BP patients or healthy controls into immune-deficient mice engrafted with human skin. Twenty-four hours after IgE injection, mice receiving BP IgE, but not control IgE, showed erythematous elevated plaques, MC activation, and dermal infiltrates of inflammatory cells. Furthermore, dermal-epidermal separation, a key clinical feature of BP, was also observed in recipient mice when a high dose of patient-derived IgE was transferred (13). This evidence from humanized mice demonstrates that IgE autoantibodies in BP patients are able to promote disease manifestation, further supporting a potential role of MCs in the pathogenesis of BP.

Besides BP, an involvement of MCs was also suggested in a range of other AIBD. For example, increased numbers of MCs have been detected in the skin of pemphigus vulgaris (PV) patients (14), in the conjunctiva of patients with ocular cicatricial pemphigoid (a subtype of MMP) (15), and in the lesional bullous skin of patients with linear IgA disease (LAD) (16). Moreover, activation of MCs has also been observed in some AIBD, including LAD (16), epidermolysis bullosa acquisita (EBA) (17), and PV (18). In addition, the presence of high concentrations of Dgs3-reactive IgE and intercellular IgE deposits in PV patients in the acute onset of the disease also indicates an involvement of MCs in PV (19).

## MCs in Mouse Models of AIBD

### Experimental Models of Autoimmune Blistering Diseases

Animal models have been established for different AIBD, such as BP, EBA, and PV *via* diverse strategies namely immunization with autoantigen, transfer of autoantibodies or autoreactive lymphocytes, and genetic modification (20). These animal models have been extensively used for investigation of disease pathogenesis (20), including the role of MCs. Here, we highlight those animal models which have been used for studying the role of MCs in AIBD (17, 21).

In 1993, Liu and his colleagues for the first time established an antibody transfer-induced mouse model for BP. By immunizing rabbits with a segment of murine COL17A homologous to the human COL17A autoantibody reactive domain, the authors generated rabbit anti-murine COL17A IgG antibodies and injected them intradermally into neonatal mice. The recipient mice developed an inflammatory blistering disease at the injection site in 2 days, providing an acute and local antibody transfer-induced model of BP. Similarly, a local antibody transfer-induced mouse model of EBA can be established, when the rabbit anti-murine COL7 IgG are transferred into mice *via* intradermal injection. In this experimental setting, IgG are injected intradermally into the base of the ear of adult mice, and the mice develop skin symptoms at the injection site within 48 h after the antibody application (17, 22). Thus, these experimental settings present acute and local antibody transfer-induced mouse models of BP and EBA.

A systemic and more chronic type of EBA can be induced in mice by repeated subcutaneous injection of rabbit anti-murine COL7 IgG directed against the pathogenic epitope of the disease (23). 4 days after the antibody injection, first disease symptoms manifest in the skin of susceptible mice, including inflammatory cell infiltration and dermal–epidermal separation. Using a similar approach, Schulze et al. established a chronic and systemic antibody transfer-induced mouse model of BP by transfer of rabbit anti-murine COL17 IgG (24).

All antibody transfer-induced mouse models for BP and EBA are characterized by infiltration of inflammatory cells into the skin, antibody, and complement deposition, and dermal–epidermal separation, representing the hallmarks of the inflammatory type of human AIBDs and make these models ideal tools to investigate the role of MCs in these diseases.

### MC-Deficient Mouse Strains

MC-deficient mouse strains are indispensable tools for investigating the role of MCs. So far, many MC-deficient strains have been reported, and those strains can be categorized into two groups according to the principle of the MC deficiency. The first group comprises KIT-dependent MC-deficient mice like WBB6F1-Kit*^W/W-v^*, WCB6F1-Mgf*^Sl/Sl-d^*, and C57BL/6-Kit*^W-sh^* mice. These mouse strains with an impaired Kit signaling due to genetic mutations affecting Kit signaling pathway. The KIT pathway is essential for the maturation and survival of MCs (4, 25). As a consequence, functional mutation within genes involved in the KIT receptor signaling could lead to the deficiency of MCs (26). However, since KIT is also expressed on many other cell types, the KIT receptor signaling deficient strains demonstrate diverse abnormalities depending on the type of genetic defects (26). The second group contains KIT-independent MC-deficient mice which are selectively deficient in MCs independent of Kit mutations, and thus without altering other parts of the immune system, such as *Cpa3^Cre/+^* knock-in mice, Mcpt5*^Cre^* transgenic mice, and Mas-TRECK mice. Among those MC-deficient mouse strains, WBB6F1-Kit*^W/W-v^*, WCB6F1-Mgf*^Sl/Sl-d^*, C57BL/6-Kit*^W-sh^*, and Mcpt5*^Cre^* transgenic mice have been used for examining the involvement of MCs in the development and progression of AIBD. An overview on the four MC-deficient mouse lines and their phenotypes are summarized in Table 1.

**Table 1:** Summary of mast cell (MC)-deficient mouse strains investigated in animal models of autoimmune bullous dermatoses (AIBD).

		Kit*^W/W-v^*	Mgf*^Sl/Sl-d^*	Kit*^W-sh^*	Mcpt5^Cre^
Abnormalities in immune system	MC deficiency	Yes	Yes	Yes	Yes^a^
	Splenomegaly	No	No	Yes	No
	Neutrophils	Decreased	–	Increased	Increased
	Basophils	Decreased	–	Increased	–
	TcRγδ intraepithelial lymphocytes	Reduced	–	Normal	Normal
	Thrombocytosis	No	No	Yes	No

Kit signaling associated abnormalities	KIT receptor signaling	Reduced	Reduced	Reduced	Not affected
	Lack of pigment	Yes	Yes	Yes	No
	Bile reflux	Yes	Yes	Yes	No
	Lack of interstitial cells of Cajal	Yes	–	Yes	No

Other abnormalities	Sterile	Yes	Yes	No	No
	Anemic	Yes	Yes	No	No
	Stomach papillomas and ulcers	Yes	Yes	No	No
	Idiopathic dermatitis	Yes	Yes	No	No
	Cardiac hypertrophy	No	–	Yes	No

Reference		(27–29)	(30–32)	(26)	(33, 34)

*–, not known*.

*^a^Deficiency in connective tissue MCs*.

#### WBB6F1-Kit^*^W/W-v^*^ Mice

The “white spotting” (*W*) locus is named after the pigment deficiency which is a result of mutations in the *Kit* gene affecting the function of melanocytes. The WBB6F1-Kit*^W/W-v^* mice are the hybrid form of WB/Re and C57BL/6 strains and carry two different types of mutations in the *W* locus, namely *W* and *W-v*. The *W* mutation, a point mutation altering a splicing site in the transcript, causes a loss of the transmembrane domain and thus hinders/impairs the cell surface expression of KIT (27). In contrast, *W-v*, a missense mutation within the KIT tyrosine kinase domain, considerably reduces the kinase activity (28). Consequently, the development of MCs in WBB6F1-Kit*^W/W-v^* mice is dramatically impaired resulting in less than 1% MCs compared to the MC-sufficient littermate controls (29). However, KIT-deficiency impairs many cell types and results e.g., in decreased numbers of neutrophils, basophils, platelets, intraepithelial TcRγδ lymphocytes, and some other KIT-associated abnormalities (29).

#### WCB6F1-Mgf^Sl/Sl-d^ Mice

WCB6F1-Mgf*^Sl/Sl-d^* mice carry two loss of function mutations, Steel (*Sl*) and Steel-Dicke (*Sl-d*) in the *Scf* gene encoding the ligand of KIT (30). The *Sl* mutation contains DNA rearrangements which lead to dysregulation of expression of the *Scf* gene (31), while the *Sl-d* mutation encodes the SCF molecule lacking the transmembrane and cytoplasmic domain (32). Combination of the two mutations dramatically impairs the production or function of SCF, and thus leading to the deficiency in the KIT signaling. As a consequence, WCB6F1-Mgf*^Sl/Sl-d^* mice show a similar pattern of abnormalities to that of WBB6F1-Kit*^W/W-v^* mice (30–32) (Table 1).

#### C57BL/6-Kit^*^W-sh^*^ Mice

The *W-sh* mutation leading to MC deficiency is an inversion located in the regulatory region upstream of the transcription start site of the *W* locus (26), affecting the expression but not changing the function of the protein. Different to Kit*^W/W-v^* mice, Kit*^W-sh^* mice are fertile and do not develop anemia, but are characterized by a proinflammatory phenotype including splenomegaly, thrombocytosis, and increased numbers of neutrophils and basophils.

#### Mcpt5^Cre^ iDTR Mice

Mast cell protease 5 (MCPT5) is a protease exclusively expressed in connective tissue MCs (CTMCs), such as peritoneal and skin MCs. In 2008, Scholten et al. generated a Mcpt5*^Cre^* transgenic mouse line expressing Cre under the control of the *Mcpt5* promoter (33). Mating this transgenic strain to other mouse lines carrying loxP-flanked genes, allows the creation of mouse lines with gene deficiency in a CTMC-specific manner. By taking this advantage, Dudeck and colleagues generated Mcpt5^Cre^ iDTR mice by crossing Mcpt5*^Cre^* with iDTR mice (34), which express a simian diphtheria toxin receptor (DTR) in loxP-flanked stop element deleted cells. In this manner, generated Mcpt5*^Cre^* iDTR mice express the DTR exclusively on CTMCs, namely peritoneal and skin MCs, which are selectively ablated by the injection of diphtheria toxin (34). This inducible MC-deficient strain develops no abnormalities in the cellular composition of spleen, blood, skin, and bone marrow (34), presenting a selective MC-deficient mouse model.

### Disease Development of Experimental AIBD in MC-Deficient Mice

Initial findings in experimental AIBD like the induction of MC degranulation after local transfer of antibodies in BP and EBA or the blockade of disease symptoms by the MC stabilizer cromolyn (17, 21) draw a high interest on the role of MC in autoantibody-mediated diseases. Consequently, pathogenesis of antibody transfer-induced AIBDs was evaluated in four different MC-deficient mouse strains. Major results were summarized in Table 2.

**Table 2:** Summary of development of experimental autoimmune bullous dermatoses (AIBD) in mast cell (MC)-deficient mouse strains.

		Antibody transfer-induced bullous pemphigoid		Antibody transfer-induced epidermolysis bullosa acquisita	
		Local model	Systemic model	Local model	Systemic model
Mice		Neonatal mice	Adult mice	Adult mice	Adult mice

Activation of mast cells (MCs)^a^		Yes	–	Yes	–

Disease development in MC-deficient mice^b^	Kit^W/W-v^	protected	–	–	susceptible, severity comparable to littermates
	Mgf*^Sl/Sl^*^-^*^d^*	Protected	–	–	–
	Kit*^W^*^-^*^sh^*	–	–	–	Susceptible, severity increased to littermates
	Mcpt5^Cre^ iDTR	–	–	Susceptible, severity comparable to littermates	Susceptible, severity comparable to littermates

Reference		(21, 37, 38)	–	(17)	(17)

*^a^Evaluated in the skin lesion of wildtype mice*.

*^b^As compared with corresponding MC-sufficient control mice*.

*–, not evaluated*.

#### Antibody Transfer-Induced BP in MC-Deficient Mice

In 2001, Chen et al. investigated for the first time MC-deficient mice in a local antibody transfer-induced mouse model of BP (21). In this study, rabbit anti-murine COL17 IgG was injected intradermally into neonatal Kit*^W/W-v^* and Mgf*^Sl/Sl-d^* mice as well as their MC-sufficient littermate controls. As expected, the MC-sufficient littermate controls developed an inflammatory skin-blistering disease, while both, Kit*^W/W-v^* and Mgf*^Sl/Sl-d^* mice, were entirely protected against the disease suggesting an indispensable role of MCs in this experimental system. Due to the numerous KIT-dependent side-effects, MC-specific effects have to be proven by further experiments, e.g., MC reconstitution in MC-deficient strains (35). Chen and colleagues could show that reconstitution of Kit*^W/W-v^* mice with bone marrow-derived MCs restored the disease confirming the essential role of MCs in this model (21). Furthermore, they found that MC activation is essential for the recruitment of neutrophils which themselves are the final executors of tissue damage (21, 36). In subsequent work, the same group could provide some information concerning the molecular mechanisms involved in MC activation in experimental BP. In 2011, they reported that Kit*^W/W-v^* mice reconstituted with C5a receptor (C5aR)-sufficient, but not C5aR-deficient MCs were susceptible to experimental BP suggesting the C5aR on MCs is critical for the development of the disease (37). Using a similar approach, they demonstrated that the role of MCs in experimental BP is also critically dependent on the MC-derived chymase MCPT4 which activates MMP9 and cleaves COL17 (38).

Taken together, the above studies employing the neonatal mouse model of antibody transfer induced BP suggesting that C5a-mediated MC activation and consequent release of MCPT4 from MCs is critical for recruitment of neutrophils from blood to the skin, and thus indicating that MCs are indispensable for the development of BP.

#### Antibody Transfer-Induced EBA in MC-Deficient Mice

Antibody transfer-induced models of BP and EBA share many clinical and histological features. Moreover, they are also comparable in pathogenesis, in terms of essential role of Fc receptors, C5a-C5aR signaling, and neutrophils (23, 39). Therefore, it is reasonable to hypothesize that MCs play an essential role in experimental EBA.

To verify this hypothesis, our group employed three different MC-deficient mouse strains to study the role of MCs in experimental EBA induced by transfer of rabbit anti-murine COL7 IgG (17). Surprisingly, when Kit*^W/W-v^* mice were used, a strain which has also been studied in BP, MC-deficient mice developed disease symptoms comparable to MC-sufficient littermate controls (17). This suggests that MCs are not required for antibody transfer-induced EBA which is in sharp contrast to their essential role in experimental BP. Moreover, repetition of this experiment in Kit*^W-sh^* mice, a further KIT-dependent MC knockout, revealed that disease developed in this strain with significant increased severity as compared to the MC-sufficient littermate controls (17). Although this increased susceptibility of Kit*^W-sh^* mice might not be due to a protective effect of MC, but more to a general proinflammatory abnormality present in this strain, these results suggest that MCs are not required for the disease manifestation in experimental EBA. Consequently, to avoid the interference from KIT signaling deficiency related other abnormalities, we investigated experimental EBA in Mcpt5*^Cre^* iDTR mice, where MC ablation was induced by treatment with diphtheria toxin. In this KIT-independent model, MC-deficient mice were susceptible to experimental disease and developed symptoms indistinguishable from those in the corresponding MC-sufficient controls. These findings derived from systemic antibody transfer-induced EBA could be reproduced in a local model induced by injection of rabbit anti-murine COL7 IgG into the base of the ear. Mcpt5*^Cre^* iDTR mice pretreated with DT also developed skin lesion at the injection site comparable to those of the MC-sufficient controls.

Taken together, these results provide strong evidence that in contrast to experimental BP, MCs are dispensable for antibody transfer-induced EBA (17).

## Conclusion and Perspectives

So far, investigations on the role of MCs have been limited to mouse models of BP and EBA and data on other AIBD are still missing. Given the similarities in clinical and pathological features between antibody transfer-induced BP and EBA, the discrepancy in the role of MCs between the two models is unexpected, but of high interest. In the last section, we will try to discuss this discrepancy and attempt to clarify the role of MCs in AIBD.

The discrepancy in the role of MCs between experimental BP and EBA may have many reasons. First of all, mice investigated in the two experimental AIBD were different; while the local model of BP was induced in neonatal mice, both local and systemic models of EBA were established in adult animals (17, 21). Since, neonatal and adult mice are different in their immune system (40, 41), this difference could also affect the role of MCs in the models. To verify this possibility, models of antibody transfer-induced BP in which adult mice are used should be employed. Second, since the role of MCs in experimental BP has been only investigated in Kit*^W/W-v^* and Mgf*^Sl/Sl-d^* mice, two strains characterized by a decreased expression of neutrophils, neutropenia, might contribute to the disease resistance as indicated for models of antibody-induced arthritis (42). This problem can be solved by investigating KIT-independent MC-deficient mice in antibody transfer-induced BP. Finally, the differences between COL17 and COL7, autoantigens of BP and EBA, respectively, might also contribute to diverging roles of MCs in both diseases.

Results derived from the different mouse models of AIBD do not provide a conclusive result on the role of MCs, more experiments need to be performed to address this issue. While it appears to be quite clear that antibody-induced EBA proceeds independently of MC, the role of these cells in corresponding models of BP needs to be reevaluated in adult KIT-independent MC-deficient mice. However, it is important to notice that each mouse model reflects only parts of the complicate entire pathogenesis of human autoimmune disease. Thus, antibody transfer-induced models mimic specifically the effector phase of inflammatory BP and EBA, and are unable to evaluate the role of MCs in the proceeding afferent phase of diseases. Since, there is evidence that MCs play a role in regulating adaptive immune responses (43), animal models mimicking both, the afferent and effector phases of AIBD, need to be used for such investigation. Furthermore, the transferred antibodies in the experimental models of BP and EBA belong to the class of IgG, and, therefore, can represent only IgG-mediated disease manifestation. Since, there is evidence that IgE autoantibodies might play a pathogenic role in BP, an animal model in which pathogenic IgE alone or in combination with IgG is transferred could address this potential key aspect in the difference between BP and EBA.

## Author Contributions

XY and FP were involved in drafting the manuscript. AK and KH were involved in correction and revision of the manuscript.

## Conflict of Interest Statement

The authors declare that the research was conducted in the absence of any commercial or financial relationships that could be construed as a potential conflict of interest.
